# Prediction of prognosis in oral squamous cell carcinoma using infrared microspectroscopy

**DOI:** 10.1002/cam4.7094

**Published:** 2024-03-12

**Authors:** Conor A. Whitley, Barnaby G. Ellis, Asterios Triantafyllou, Philip J. Gunning, Peter Gardner, Steve D. Barrett, Richard J. Shaw, Caroline I. Smith, Peter Weightman, Janet M. Risk

**Affiliations:** ^1^ Department of Physics University of Liverpool Liverpool UK; ^2^ Department of Pathology, Liverpool Clinical Laboratories University of Liverpool Liverpool UK; ^3^ Department of Molecular and Clinical Cancer Medicine Institute of Systems, Molecular and Integrative Biology, University of Liverpool Liverpool UK; ^4^ Department of Chemical Engineering The University of Manchester Manchester UK; ^5^ Regional Maxillofacial Unit Liverpool University Hospitals NHS Foundation Trust Liverpool UK

**Keywords:** biomarker, FTIR, infrared microspectroscopy, OSCC, prognosis

## Abstract

**Background:**

Estimation of prognosis of oral squamous cell carcinoma (OSCC) is inaccurate prior to surgery, only being effected following subsequent pathological analysis of the primary tumour and excised lymph nodes. Consequently, a proportion of patients are overtreated, with an increase in morbidity, or undertreated, with inadequate margins and risk of recurrence. We hypothesise that it is possible to accurately characterise clinical outcomes from infrared spectra arising from diagnostic biopsies. In this first step, we correlate survival with IR spectra derived from the primary tumour.

**Methods:**

Infrared spectra were collected from tumour tissue from 29 patients with OSCC and subject to classification modelling.

**Results:**

The model had a median AUROC of 0.89 with regard to prognosis, a median specificity of 0.83, and a hazard ratio of 6.29 in univariate Cox proportional hazard modelling.

**Conclusion:**

The data suggest that FTIR spectra may be a useful early biomarker of prognosis in OSCC.

## Graphical abstract

Estimation of prognosis of oral squamous cell carcinoma (OSCC) is inaccurate prior to surgery leading to over treatment in a proportion of patients. Infrared spectra were collected from OSCC tumour tissue and subject to classification modelling. The model had a median AUROC of 0.89 with regard to prognosis, a median specificity of 0.83 and a hazard ratio of 6.29 in univariate Cox proportional hazard modelling. The data suggest that FTIR spectra may be a useful early biomarker of prognosis in OSCC.

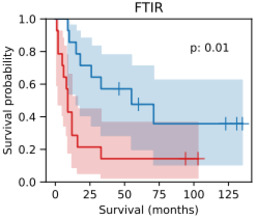

## 1 INTRODUCTION

Oral squamous cell carcinoma (OSCC) is the eighth most common form of cancer in the United Kingdom,^1^ and the incidence continues to increase.^2^ Therapy is primary surgical ablation, reconstruction as required, and adjuvant radiotherapy or chemoradiotherapy tailored to pathological analysis and staging of the resection specimen. Pathological analysis also allows identification of lymph node metastases and metastases beyond the contour of the node (extra‐nodal extension, ENE), which are of prognostic importance.^3^, ^4^ For cases with lower biological aggression, de‐escalation of treatment may be possible.^5^, ^6^ Furthermore, early identification of patients with adverse prognosis despite comprehensive treatment could allow targeting for neo‐adjuvant therapy or for inclusion into clinical trials including immunotherapy and other novel agents. Prediction of poor prognosis could also allow more comprehensive lymph node clearance or surgical margins. However, estimation of prognosis prior to surgery is a significant problem, as pathological staging of the margin, characteristics of grading, or cervical lymph node metastases are not yet available.

Cross sectional imaging using magnetic resonance (MRI) and computed tomography (CT) are of limited value in identifying ENE^7^ despite improvements in imaging analysis utilising deep learning models.^8^ It has been hypothesised that tumours with ENE may carry a distinct molecular fingerprint, the identification of which would allow for targeting of patients towards appropriate treatment.^9^, ^10^ However, a search for markers of poor prognosis that can be utilised prior to primary treatment has not yet been successful.

Of alternative technologies considered, Fourier‐transform infrared (FTIR) microspectroscopy has been recently utilised in a range of biomedical settings.^11^, ^12^, ^13^ This technology allows for imaging of sample specimens at thousands of infrared (IR) wavelengths simultaneously. Spectral differences from biochemical compounds are typically located in a region known as the fingerprint region (1000–1800 cm^−1^) and it is differences in absorption at these wavenumbers which contain information that can be utilised to discriminate between areas of interest in samples. For example, a preliminary study indicated that FTIR spectroscopy can discriminate samples of epithelial oral dysplasia lesions that later transform from those that do not.^14^

The present investigation explores the potential efficacy of FTIR microspectroscopy as a method of identifying OSCC patients with poor prognosis prior to surgery.

## 2 MATERIALS AND METHODS

### 2.1 Samples

A previously described tissue microarray (TMA) prepared from 102 patients with OSCC^15^ and consisting of 1 mm diameter, formalin fixed, paraffin embedded (FFPE) tissue cores was utilised to prepare samples for FTIR analysis. Patients had given written, informed consent, the study design conforms to the principles of the Declaration of Helsinki and was undertaken under ethical approval (Northwest – Liverpool Central REC number EC47.01). Four adjacent sections of 4 μm thickness were cut from the TMA; the first and last sections were routinely stained with haematoxylin and eosin (H&E) to assess the presence and extent of tumour. Images of the stained sections were scanned using an Aperio CS2 scanner (Leica Biosystems) and used for IR image annotation. The second and third TMA sections were mounted onto CaF_2_ disks and used for FTIR microspectroscopy.

The TMA had been previously sectioned and some cores were depleted of tumour, so selection criteria for this study were a diagnosis of OSCC; the presence of OSCC in the TMA core; the ability to co‐register adjacent H&E‐stained and FTIR‐imaged sections and a follow‐up period after surgery of at least 24 months.

### 2.2 Risk stratification

The peak time for OSCC tumour recurrence is 9 months post surgery with most of the recurrences and deaths occurring by 2 years.^16^ In order to identify a survival endpoint that characterised prognosis for this cohort, patients were stratified into high‐ and low‐risk categories based on outcome data alone using an optimisation routine which maximised the log‐rank statistic with respect to the patient groupings^17^ (see Appendix S1). At the maximum log‐rank score, the time point that separated the high‐risk group of patients from those at low‐risk was used as the survival cut‐off in subsequent risk modelling.

### 2.3 FTIR microspectroscopy

FTIR measurements of the TMA cores were taken as described in Appendix S1. Images were acquired at a resolution of 6 cm^−1^ over a spectral range of 990–3800 cm^−1^. Background scans were acquired using a blank CaF_2_ disk. Data were extracted from raw output using MATLAB methods from ChiToolBox.^18^

### 2.4 Data preprocessing and analysis

Areas consisting largely of tumour were identified on the scanned H&E images and these were co‐registered with IR images at 1650 cm^−1^ (the amide‐I peak) from the same tissue core to identify relevant IR data for analysis. Corrections for atmospheric scattering, sample thickness, paraffin contamination and poor‐quality spectra were undertaken (see Appendix S1) and spectra within the fingerprint region (1000–1800 cm^−1^) were extracted for classification modelling.

Scientific Python packages (SciPy)^19^, ^20^, ^21^ were utilised for classification modelling and survival analysis. Seven principal components of the data were taken to reduce data complexity prior to the logistic regression (LR) classifier. The classification power of an FTIR spectrum as a prognostic biomarker was estimated using area under the receiver operating characteristic curve (AUROC), specificity and sensitivity. To estimate the variability of the classification power of FTIR, bootstrap out‐of‐bag sampling was utilised (see Appendix S1). The linear regression (LR) model gave the risk group classification for each datapoint as a probability score. The final prediction scores for each patient (the patient prediction score) were defined by the median probability for all datapoints for that patient. Prognostic efficacy was investigated using Kaplan–Meier survival analysis, a Cox proportional hazards regression and a log‐rank test. Confidence intervals for the Kaplan–Meier analysis were computed using the exponential Greenwood method.^22^

## 3 RESULTS

The selection criteria resulted in a cohort of 29 patients that reflected the original patient set^15^ in most regards (Table S1).

**FIGURE 1:**
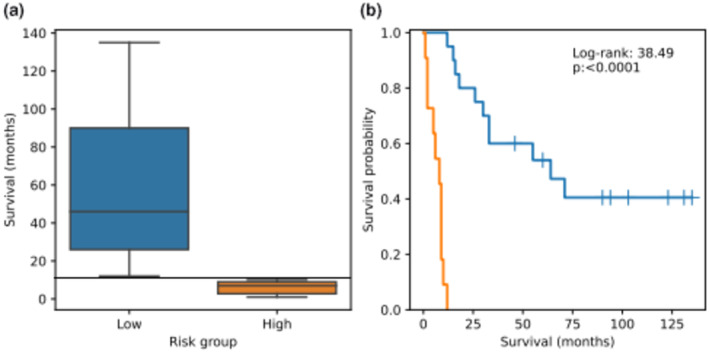
Stratification of patients into low‐ and high‐risk groups. (A) Whisker box plots of survival duration in months for low‐ (left) and high (right)‐risk groups. Horizontal dotted line indicates the survival time that separates the two groups; (B) Kaplan–Meier survival curves of each risk group. The log‐rank statistic and corresponding *p*‐value are also shown.

Risk stratification established that a time point of 11 months separated the high‐risk group of patients from those at low‐risk (Figure 1A) and the two groups showed a clear distinction in outcome (Figure 1) using this cut‐off. Survival at 11 months was used as the delimiter in subsequent risk modelling.

**FIGURE 2:**
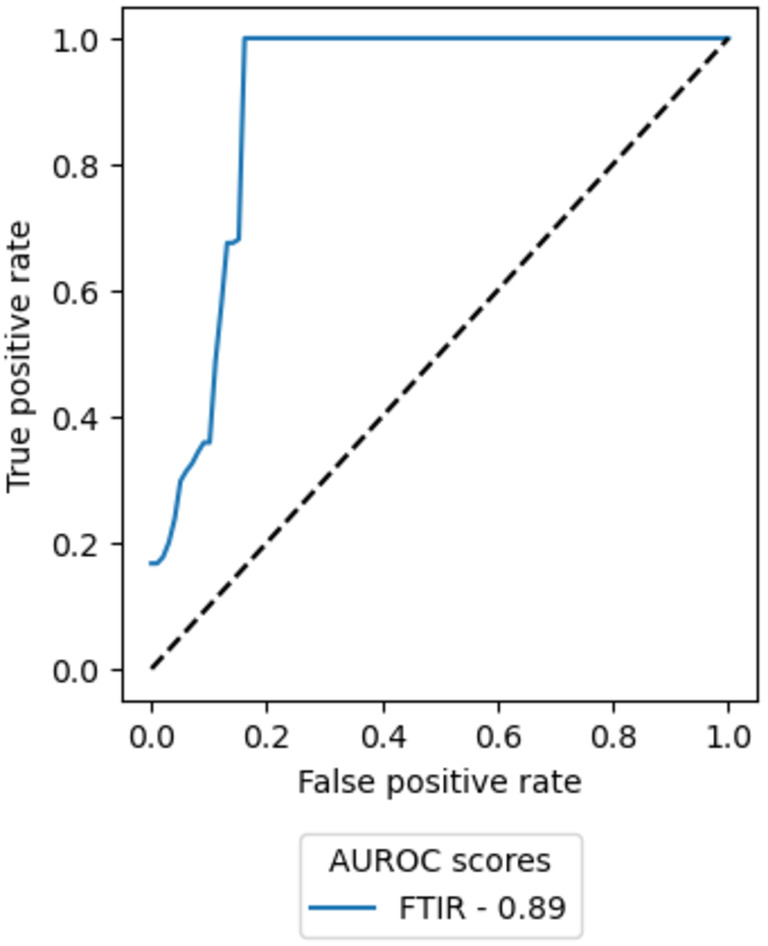
Prediction of risk of death within 11 months using patient prediction scores. Median AUROC curve for the FTIR‐based model. The dashed black lines represent baselines scores associated with random chance.

The classification power of the FTIR spectra as a biomarker of prognosis for each individual patient had a median sensitivity of 1.0 (±0.04), a median specificity of 0.83 (±0.04) and a median AUROC of 0.89 (Figure 2). A univariate Cox proportional hazard model was fit to patient prediction scores to assess the prognostic utility of the prediction scores and gave a hazard ratio of 7.516 (95% CI: 1.116–50.62; *p* = 0.04).

**FIGURE 3:**
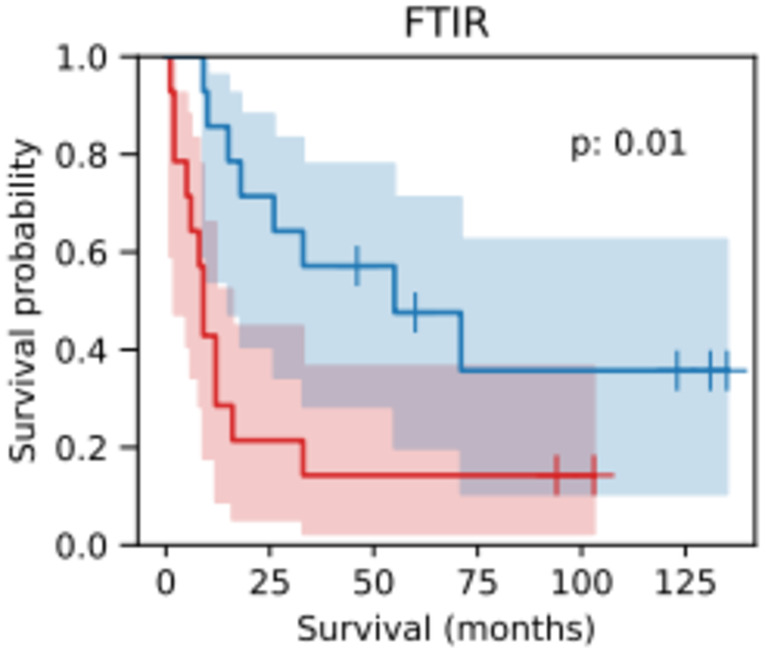
Kaplan–Meier survival curves for each risk group. Blue line: low‐risk patient group defined by the model; red line: high‐risk patient group defined by the model. Shading indicates confidence intervals.

In a reverse analysis, the classification threshold from the model that maximised the log‐rank statistic was used to give a binary assignment of risk by re‐assigning patients to high‐ or low‐risk groups and survival analysis was undertaken. Significant separation of the risk groups was obtained (Figure 3; *p* = 0.01).

## 4 DISCUSSION

Despite the accepted premise that tumours with poor prognosis will demonstrate molecular differences, a search for markers of poor prognosis that can be utilised prior to primary treatment has not yet been successful. As a first step to explore the potential efficacy of FTIR microspectroscopy as a method of identifying OSCC patients with poor prognosis prior to surgery, the present investigation suggests that the chemical ‘fingerprint’ of the primary tumour, as determined using FTIR microspectroscopy, is able to identify individuals whose disease will adversely progress following treatment.

The patient cohort in this study was a subset of that in a previous study of prognostic indicators in OSCC.^15^ However, owing to extensive prior sectioning of the TMA used in that study and the selection criteria applied here, which required co‐registration between sections stained with H&E and those used to collect FTIR data, the cohort size was reduced. Nevertheless, classification statistics of the model utilising FTIR data were generally high, with a median AUROC of 0.89, median specificity of 0.83, median sensitivity of 1.0, and a univariate Cox proportional hazard model produced a hazard ratio of 7.52. Taken together, these data suggest that FTIR spectra are a prognostically useful biomarker. As with all such modelling, there is a trade‐off between sensitivity and specificity and the selection of an appropriate model threshold value for application in a clinical setting will depend on the clinical question under consideration. If FTIR data were to be used to analyse OSCC diagnostic biopsies with a view to stratification for trials, for example, of window I/O agents, then specificity will be the most valuable classification statistic and the model can be adjusted to provide the best value for this statistic.

It has previously been established that the presence of ENE is the most predictive of several staging and grading markers of prognosis in oral cancer and is a better predictor than nodal status alone.^3^, ^4^ In our cohort, the sensitivity and specificity for ENE as a predictor of death from disease by 12 months was inferior, 0.60 and 0.78, respectively, to the FTIR model. Comparisons with other clinicopathological markers of prognosis such as PNI (perineural invasion), margin status and tumour thickness in our cohort again show that FTIR data is superior as a prognosticator. Moreover, as with ENE, these indicators cannot be used to estimate prognosis prior to surgery. Immunohistochemical and molecular analyses to identify putative biomarkers of ENE in diagnostic biopsy material have suggested that the presence of cancer associated fibroblasts (CAFs) may be a marker of aggression.^23^, ^24^ However, the hazard ratio obtained from FTIR modelling in the current paper is considerably better than those obtained when the CAF marker, *α*SMA, is used to predict shortened survival times.^23^, ^24^ Furthermore, MRI is of limited use in determining nodal status of OSCC prior to surgery (sensitivity 0.56; specificity 0.84 in our cohort^15^) or as a prognostic indicator (sensitivity 0.46; specificity 0.65 in our cohort) and is inadequate for the pre‐surgery detection of ENE,^7^ identifying less than 3% of cases in our cohort.^15^

The 95% confidence intervals for the hazard ratio obtained using FTIR data spanned a large range, indicating a high degree of heterogeneity within the dataset and would justify exploration of a larger cohort in future. This spread may be attributed to the small sample size of 29 patients as, no matter how carefully selected the study cohort is, there will be some differences in tumour characteristics and in treatment plans between patients. Alternatively, the range of the 95% CI may be due to the heterogeneity of tumour cell populations.^25^, ^26^ The size of each FTIR datapoint (i.e. the resolution) in this study is of the order of 10 μm, similar to the size of individual cells, but it is highly unlikely that all cells within a tumour will have the same biological potential. During our analysis, each datapoint (equivalent to a single cell) within each sample would have been associated with the survival data from the whole patient during the analysis. Thus, there will be some underlying mismatch of individual datasets and survival data.

Taken together, these data agree with a recent meta‐analysis suggesting that the use of machine learning to analyse large datasets related to molecular markers produces better models than when machine learning is not used, or in models that do not incorporate such markers.^27^ It is interesting to speculate what biochemical compounds in the tumour the discriminating FTIR wavenumbers represent. Given the results of two recent meta‐analyses demonstrating that the presence of cancer‐associated fibroblasts in OSCC are a negative prognostic indicator,^23^, ^25^ it might be hypothesised that part of the FTIR ‘signature’ will identify markers of these cells. This hypothesis would require further validation, characterising the wavelengths that constitute most of the weighting and their correlating cellular or molecular characteristics. Furthermore, the applicability of the technique to pre‐surgical, diagnostic biopsy specimens is not yet proven. Although a recent study of the adequacy of biopsy specimens for prognosis suggested that they did not reflect the commonly used clinical prognostic feature of tumour depth,^28^ it is possible that these specimens would be sufficient for FTIR‐based prognostication.

The ability to identify patients with poor prognosis prior to surgery would provide the opportunity to change the management of such patients. For example, as a stratification factor for a novel treatment modality such as induction immune‐oncology; or as an indicator of the need to intensify the treatment plan by the addition of chemotherapy and/or the intensification of radiotherapy. It is also conceivable, that additional analysis of the ‘adverse chemical signature’ will identify a novel therapeutic target.

## 5 CONCLUSION

Exploring possible clinical applications of FTIR is a growing field, but little attention has been paid towards its use as a prognostic biomarker in OSCC. Similarly, the use of machine learning algorithms to assess clinical or other biomarker data to predict survival has been developing rapidly and is gaining recognition.^29^ The present study suggests that FTIR microspectroscopy has the potential to augment prognostic predictive capabilities. A larger study is required to precisely evaluate the present findings; reduce the effect of tumour heterogeneity on the analysis; and investigate the utility of this technique on diagnostic biopsies, rather than resection specimens.

## AUTHOR CONTRIBUTIONS

**Conor A. Whitley:** Conceptualization (supporting); data curation (lead); formal analysis (lead); investigation (lead); methodology (equal); visualization (equal); writing – original draft (lead); writing – review and editing (equal). **Barnaby G. Ellis:** Formal analysis (supporting); investigation (supporting); methodology (supporting); writing – review and editing (supporting). **Asterios Triantafyllou:** Formal analysis (supporting); investigation (equal); methodology (supporting); resources (equal); supervision (supporting); writing – original draft (supporting); writing – review and editing (equal). **Philip J. Gunning:** Investigation (supporting); resources (equal); writing – review and editing (supporting). **Peter Gardner:** Formal analysis (supporting); resources (equal); software (equal); writing – review and editing (equal). **Steve D. Barrett:** Data curation (equal); formal analysis (equal); funding acquisition (equal); methodology (equal); supervision (equal); writing – review and editing (equal). **Richard Shaw:** Conceptualization (supporting); formal analysis (supporting); funding acquisition (equal); supervision (supporting); writing – original draft (supporting); writing – review and editing (supporting). **Caroline I. Smith:** Data curation (equal); formal analysis (supporting); project administration (supporting); resources (supporting); writing – review and editing (supporting). **Peter Weightman:** Formal analysis (equal); funding acquisition (lead); project administration (supporting); supervision (lead); writing – original draft (equal); writing – review and editing (equal). **Janet M. Risk:** Conceptualization (lead); formal analysis (supporting); funding acquisition (lead); investigation (lead); methodology (supporting); project administration (lead); resources (lead); supervision (equal); visualization (equal); writing – original draft (equal); writing – review and editing (lead).

## FUNDING INFORMATION

This study was funded by Cancer Research UK C7738/A26196. BGE and CAW were supported by Engineering and Physical Sciences Research Council (EPSRC) PhD studentships.

## CONFLICT OF INTEREST STATEMENT

The authors assert that they have no conflict of interest to declare.

## ETHICS STATEMENT

This study was undertaken under ethical approval (Northwest – Liverpool Central REC number EC47.01). All patients gave informed, signed consent to take part in this study.

## Supporting information


Appendix S1

## Data Availability

The data that support the findings of this study are available from Dr Caroline Smith (cismith@liverpool.ac.uk) upon reasonable request.
