# Supplementary material for: Prediction of prognosis in oral squamous cell carcinoma using infrared microspectroscopy
Source: Cancer Med. 2024 Mar 12;13(5):e7094. doi: 10.1002/cam4.7094 (PMC10928453; doi:10.1002/cam4.7094)
Supplement: Supplementary file 1 — Appendix S1 [file CAM4-13-e7094-s001.docx]

**Supplementary Information: Methods**

*Risk stratification*

In the genetic algorithm (GA)-based approach to risk grouping optimisation utilising the distributed evolutionary algorithms for python (DEAP) library, ‘individuals’ were vectors comprising the identity of the risk grouping of each patient in the analysis at a given time point. The ‘fitness’ of each individual was defined as the log-rank statistic and was calculated in comparison to the actual patient risk grouping specified by survival of that individual at that time point. Multiple solutions with similar log rank scores were obtained, but the solution with the highest log rank statistic was selected and used to create Figure 1 in the main paper. (Supplementary figure 1)


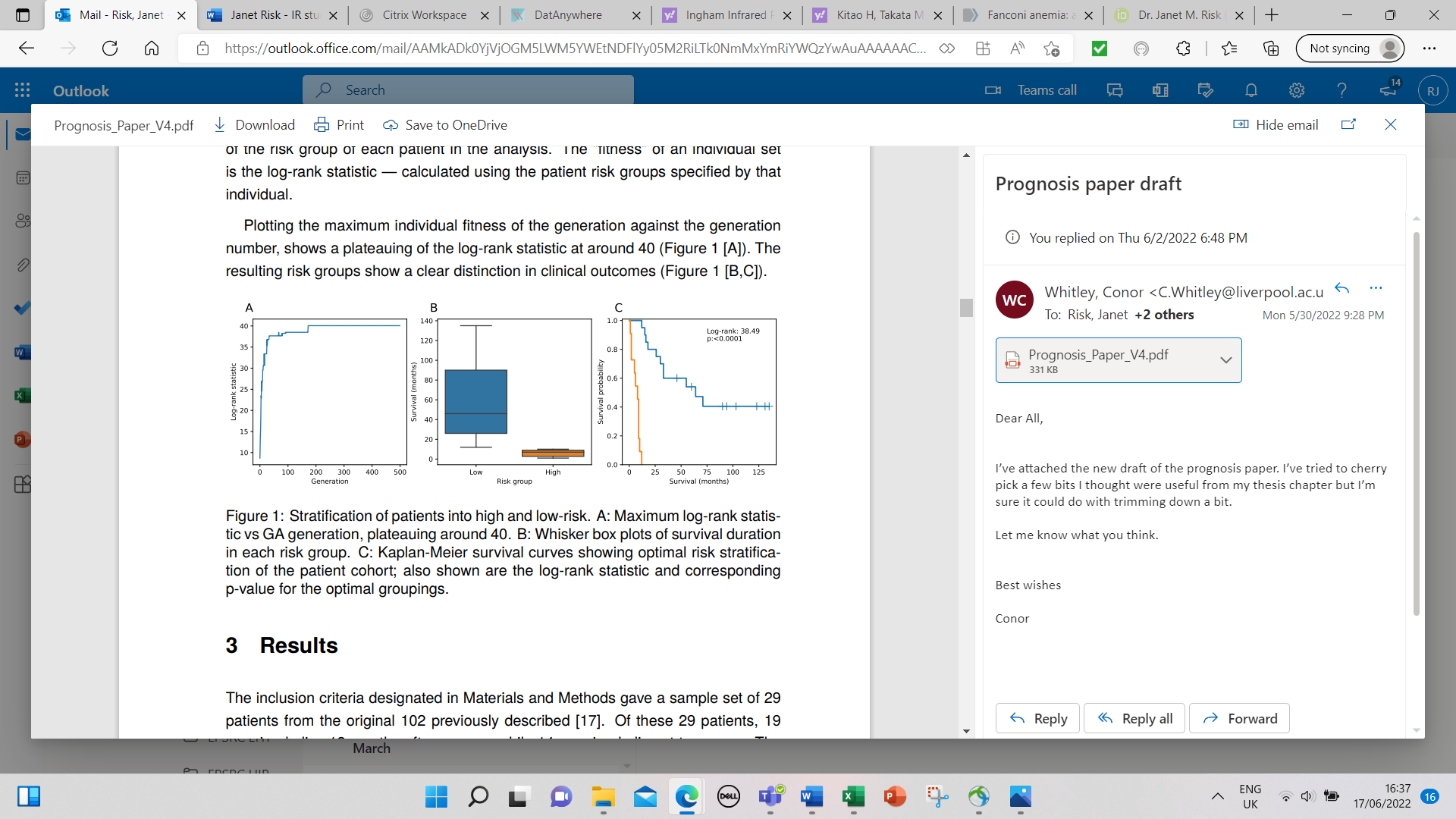


**Supplementary Figure 1**: Maximum log-rank statistic vs genetic algorithm (GA) generation

*FTIR Microspectroscopy*

FTIR measurements of the TMA cores were taken at room temperature (~20±1 ̊C) under conditions of low humidity (<1%) using a Varian Cary 670-FTIR spectrometer with an attached Varian Cary 620-FTIR microscope (now Agilent Technologies, Santa Clara CA, USA) with a liquid nitrogen-cooled 128×128 pixel mercury cadmium telluride (MCT) focal plane array and an effective field of view for each pixel of 5.5 *μ*m. Images were acquired at a resolution of 6 cm^−1^ over a spectral range of 990 cm^−1^ to 3800 cm^−1^ using a co-addition of 128 scans. Background scans were acquired using a blank CaF_2_ disk. Data was extracted from raw output using MATLAB methods from ChiToolBox^18^.

*Data pre-processing and analysis*

To correct for atmospheric scattering, extracted spectra were pre-processed using an open-source extended multiplicative scattering correction (EMSC) code^S1^. Multivariate Hotelling’s *T*^2^ statistic was used as an unsupervised quality control check of all data to eliminate anomalous spectra^S2,S3^; spectra with a *T*^2^ value lying outside the 95*th* percentile were deemed to be anomalous and were omitted from further analysis. The absorbances situated in the range of 1340cm^−1^-1460cm^−1^, attributable to paraffin, were removed. Vector normalisation was used to account for sample thickness and wavenumber absorbance features were mean-centred with variance scaled to one.

A large L2 regularisation term (1 × 10^5^) corresponding to the sum of the squares of the feature weights was applied to the objective function when fitting the LR model to mitigate the potential for overfitting. When fitting the LR model, data points were inversely weighted to compensate for the differing number of acquired spectra per patient and by risk group to mitigate the imbalanced nature of the dataset.

Bootstrap out-of-bag sampling: This process was applied to estimate the variability of the risk model and was undertaken after the model had assigned patients to FTIR data-based risk groups. A training dataset was constructed by drawing FTIR data from 80% of patients in the total dataset (23/29). The remaining 20% of data (6/29 patients) was used as the ‘out of bag’ test set on which the fitted model was evaluated. Classification statistics (AUROC, sensitivity, specificity) were calculated from each iteration. This process was repeated 100 times, ensuring that no two sample set combinations were identical, and the means/medians of the classification statistics were used to describe the model.

**References**

[S1] Köhler A et al. Estimating and correcting Mie scattering in synchrotron-based microscopic fourier transform infrared spectra by extended multiplicative signal correction. Applied Spectroscopy 2008;62:259–266.

[S2] Pitard FF. An Introduction to the Theory of Sampling: An Essential Part of Total Quality Management. Comprehensive Chemometrics 2009;1:1–16.

[S3] Härdle WK, Simar L. Applied multivariate statistical analysis, fourth edition. 2015, pp. 1–580.

[S4] Rogers SN, et al. Survival following primary surgery for oral cancer. Oral Oncology 2008;45:201–211.

Supplemental Table 1: Clinicopathological features.

| **Feature** | **Current cohort (n=29)** | **Original cohort^15^**  **N=102** | **Larger, local cohort^S4^**  **N=489** |
| --- | --- | --- | --- |
| **Age (years)** |  |  |  |
| Mean  Range  Median | 60  29-85  61 | 60  29-89 |  |
| **Sex** |  |  |  |
| F  M | 7 (24)*  22 (76) | 57 (56)  45 (45) | 187 (38)  302 (62) |
| **Site** |  |  |  |
| Floor of mouth  Tongue  Other | 8 (28)  9 (31)  12 (41) | 35 (34)  37 (36)  24 (24) | 162 (33)  144 (30)  183 (36) |
| **pT** |  |  |  |
| 1 + 2  3 + 4 | 15 (52)  14 (48) | 63 (62)  39 (38) | 293 (61)  191 (39) |
| **pN** |  |  |  |
| 0  1  2 | 7 (24)  7 (24)  20 (69) | 38 (38)  18 (18)  45 (45) | 314 (65)  64 (13)  101(21) |
| **ENE** |  |  |  |
| No  Yes | 13 (45)  16 (55) | 61 (60)  41 (40) | 388 (79)  101 (21) |
| **Margins**  Clear  Close  Involved | 6 (26)  11 (48)  6 (26) | 26 (32)  37 (45)  19 (23) | 237 (48)  170 (35)  82 (17) |
| **Tumour thickness**  ≤2cm  ≥2cm and ≤4cm  >4cm | 14 (67)  7 (33) | 63 (82)  13 (17)  1 ( 1) | 116 (24)  224 (47)  137 (29) |
| **PNI**  Absent  Present | 14 (64)  8 (36) | 50 (66)  26 (34) | 364 (74)  125 (26) |
| **RT**  No  Yes | 6 (21)  23 (79) | 31 (31)  70 (69) | 295 (60)  194 (40) |
| **Survival** |  |  |  |
| Alive at 12 months:  No  Yes | 9 (31)  20 (69) | 25 (25)  76 (75) | ND |
| Alive at 24 months:  No  Yes | 15 (52)  14 (48) | 38 (38)  63 (62) | 252 (52)  237 (48) |

TNM Atlas, 7^th^ Edition was used in pathology reporting of this cohort.

*: numbers in parenthesis are percentages; ND = not determined
